# ALK Status Assessment with Liquid Biopsies of Lung Cancer Patients

**DOI:** 10.3390/cancers9080106

**Published:** 2017-08-12

**Authors:** Paul Hofman

**Affiliations:** 1 Laboratory of Clinical and Experimental Pathology, Côte d’Azur University, FHU OncoAge, 06001 Nice Cedex 01, France; hofman.p@chu-nice.fr; Tel.: +33-4-9203-8855; Fax: +33-4-9203-8850; 2 Hospital-Integrated Biobank (BB-0033-00025), Pasteur Hospital, 30 Avenue De La Voie Romaine, 06001 Nice Cedex 01, France

**Keywords:** *ALK*, rearrangement, mutations, lung cancer, liquid biopsy, circulating tumor cells, platelets, plasma

## Abstract

Patients with advanced stage non-small cell lung carcinoma (NSCLC) harboring an anaplastic lymphoma kinase *ALK* gene rearrangement, detected from a tissue sample, can benefit from targeted ALK inhibitor treatment. However, while treatment is initially effective in most cases, relapse or progression occurs due to different resistance mechanisms including mutations in the tyrosine kinase domain of echinoderm microtubule-associated protein-like 4 (EML44)-ALK. The liquid biopsy concept has recently radically changed the clinical care of NSCLC patients, in particular for those harboring an epidermal growth factor receptor (*EGFR*) gene mutation. Therefore, liquid biopsy is an alternative or complementary method to tissue biopsy for the detection of some resistance mutations in *EGFR* arising during tyrosine kinase inhibitor treatment. Moreover, in some frail patients, or if the tumor lesion is not accessible to a tissue biopsy, a liquid biopsy can also detect some activating mutations in *EGFR* on initial assessment. Recent studies have evaluated the possibility of also using a liquid biopsy approach to detect an *ALK* rearrangement and/or the emergence during inhibitor treatment of some resistance mutations in *ALK*. These assessments can be performed by studying circulating tumor cells by fluorescent in situ hybridization and by immunocytochemistry and/or after the isolation of RNA from plasma samples, free or associated with platelets. Thus, the liquid biopsy may be a complementary or sometimes alternative method for the assessment of the *ALK* status in certain NSCLC patients, as well as a non-invasive approach for early detection of *ALK* mutations. In this review, we highlight the current data concerning the role of the liquid biopsy for the *ALK* status assessment for NSCLC patients, and we compare the different approaches for this evaluation from blood samples.

## 1. Introduction

Echinoderm microtubule-associated protein-like 4- anaplastic lymphoma kinase *EML4-ALK* gene rearrangements are present in around 5% of non-small cell lung carcinoma (NSCLC) patients [1,2]. In the majority of cases, they are adenocarcinomas [1,2]. It is noteworthy that, in some series, more than 17% of lung cancer patients who never smoked have this genomic alteration [1]. Currently, analysis for the *EML4-ALK* status is mainly performed by immunohistochemistry (IHC) and confirmed by FISH analysis from tissue biopsies [3,4]. This status can also be detected on cytological samples by immunochemistry (ICC) and/or by FISH analysis [5,6,7,8,9]. However, molecular biology methods are also used for this genomic alteration detection in tissues or cells, in particular by next generation sequencing (NGS) [10,11]. The presence of an *EML4-ALK* rearrangement leads to targeted treatment [12]. Despite a frequent early response, relapse and progression is systematically noted, which can lead to a change in targeted therapy [13,14]. In most cases, this tumor progression is linked to the emergence of *EML4-ALK* mutations [15].

The development of liquid biopsies (LB) has changed substantially the care of patients with solid cancers, in particular late stage lung cancer patients [16,17,18,19]. Addition of LB to management of advanced lung cancer can have a strong impact at three different levels: (i) for the initial detection of actionable oncogenic drivers; (ii) for the identification of resistance mutations in patients relapsing on targeted therapies; and (iii) for the monitoring of the response to therapy and the prediction of the clinical outcome [20,21,22,23,24,25,26,27]. Within the context of targeted therapeutics, the question arises of whether to use LB on a daily basis to evaluate the presence of rearrangements or mutations in *ALK* and to propose this approach instead of an analysis made from tissue and/or cytological samples.

This review will briefly discuss the notion of LB and then analyze the advantages and limitations of the different methods that evaluate the *ALK* status using blood from patients with advanced stage lung cancer, performed either at the time of diagnosis or during follow-up of the patient on targeted treatments. 

## 2. The Different Biological Components of a Liquid Biopsy

LB contains several components of interest for research into potential therapeutic targets [28,29]. Two main components have been studied, circulating tumor cells (CTCs) and plasma [28,29]. At present, most of the approaches in routine practice use plasma, in particular for activating and resistance mutations detection in EGFR in patients with advanced stage lung cancer. 

Many direct and indirect methods have been developed to detect and characterize CTCs associated with lung cancer [30,31]. These different methods have advantages and disadvantages [30,31]. Different innovative technologies to improve the detection and characterization of CTCs in NSCLC have been developed, including CTC microchips, filtration devices, quantitative reverse-transcription PCR assays, and automated microscopy systems [30,31]. Direct approaches are based on the isolation of CTCs through their physical characteristics [30,31]. Thus, in these latter methods, the CTCs are detected through their different size, their loss of deformability, their higher density and their electrical charges [30,31]. Indirect techniques exploit the molecular properties of CTCs [30,31]. Among these latter methods, the only FDA approved method for CTC detection, the CellSearch method (Janssen Diagnostics Company, Raritan, NJ, USA), has been FDA approved for CTC detection in metastatic breast, prostate and colon cancer patients. However, the CellSearch method has not been approved for CTC detection in metastatic lung cancer patients by the FDA. It is noteworthy that, since CTCs that have undergone epithelial-mesenchymal transition cannot express epithelial biomarkers, the CellSearch system can miss the detection of a number of CTCs of interest in lung cancer patients [30,31]. Currently, the results obtained from CTCs characterization do not change the treatment of patients on a daily practice. Thus, to date, studies into the detection and characterization of CTCs are still a matter of translational and clinical research in NSCLC patients. The methods used to isolate free plasma DNA can also isolate circulating tumor DNA, which is present in variable amounts depending on the cancer patients and tumor stage. The volume of whole blood used is generally 10 mL but a larger volume is sometimes necessary for certain analyses, in particular NGS. The analyses are either targeted techniques such as allele specific PCR or droplet digital PCR or NGS techniques using panels of different genes. The sequenom mass array approach can also detect genomic alterations on several genes in blood samples [32,33]. At present, the detection of activating or resistance mutations in *EGFR* in plasma samples can be performed in the clinical care of patients with advanced stage lung cancer according to the different practices available.

Other components of blood can be used for detection of some therapeutic targets for patients with lung cancer, including free plasma RNA or RNA in a complex with proteins in exosome or in association with platelets. Among these RNA, microRNA are of particular interest. Microparticles in plasma are heterogeneous components associated with exosomes, nucleosomes and particles of larger size. However, currently, none of these elements are used in a routine manner for detection of genomic alterations in lung cancer patients.

## 3. Analysis of the ALK Status with Circulating Tumor Cells

Several recent studies showed that EML4-ALK rearrangement could be detected within CTCs [34,35,36,37,38,39,40]. In 2012 a study from our group showed very good agreement between the presence of *EML4-ALK* rearrangements in tissue biopsies and in CTCs [37]. In this study, detection of CTCs was performed with a direct method (ISET) using filtration of blood through a porous filter which retained on its surface CTCs that are larger than circulating hematological cells. The ISET method used in this work has the advantage of being able to characterize the cyto-morphology of the isolated CTCs and the malignant cytological criteria can be used to identify the different populations of cells on the surface of the filter [41]. Moreover, this approach holds the advantage of allowing correlation between the ALK status, determined by ICC and/or FISH and the malignant cytological characteristics which limits potential false positive results. In this context, since it was reported that ICC and FISH analyses gave the same results, the question was to know if ICC can be used for systematic rapid screening of *ALK* status in isolated CTCs. In fact, ICC results can be easily interpreted by cytopathologists in any pathology laboratory and performed in an identical way to detection of ALK rearrangements using tissues or cells. However, despite the promising results from the initial study, one limitation was that only a small number of patients at a single center were studied [37]. In this regard, a French multi-center study with a large number of patients with a tissue biopsy *ALK* positive status is now in progress [42] (www.clinicaltrials.gov/ct2/show/NCT02372448). Another limitation of the initial study was that no information existed concerning a possible association between targeted treatments for patients presenting with *ALK* positive CTCs and the therapeutic response, in particular with respect to the CTC number. Finally, some patients often had less than 100 analyzable CTCs, which make the interpretation of a positive score difficult to take into consideration. A second study using the ISET method also showed a correlation between the presence of an *EML4-ALK* rearrangement in tissues and CTCs and a correlation with the disappearance of CTCs with a good response to targeted therapy [38]. The analysis with immunofluorescence and confocal microscopy demonstrated that CTCs harboring a unique *ALK* rearrangement may have a mesenchymal phenotype with the potential to drive metastatic progression of *ALK*-positive NSCLC [38]. Using the same ISET method for CTCs detection, a recent study looked for the prognostic value of patients with CTCs having *ALK*-copy number gain (CNG) at baseline and treated with crizotinib [39]. In this study, the dynamic change in the numbers of CTCs with *ALK*-CNG was a predictive biomarker for crizotinib efficacy in *ALK*-rearranged NSCLC patients [39]. Another study confirmed the correlation between a positive *EML4-ALK* status detected in tissues and in CTCs detected by FISH analysis [40]. This latter study showed that persistence of or an increase in the number of *ALK* positive CTCs on FISH analysis correlated with tumor progression in patients on treatment with crizotinib, in particular with the appearance of a new molecular profile [40]. Finally, a recent work demonstrated that ALK-rearrangement can also be detected in CTCs collected from advanced NSCLC patients by another approach, the NanoVelcro system [36].

## 4. Analysis of ALK Status with Plasma

Free plasma DNA is routinely used to analyze mutations in *EGFR* in patients with advanced stage lung cancer. In contrast, plasma RNA is not used routinely, or very rarely used, for detection of *EML4-ALK* rearrangements. Nonetheless, the detection of this genomic alteration can be performed with plasma using the reverse transcription-polymerase chain reaction (RT-PCR). While in theory this approach can detect an *ALK* rearrangement in the blood of patients shown to be *ALK* positive after analysis of a tissue biopsy, it still has some limits. The first hurdle concerns mastering of the pre-analytical delay between the blood sampling and the extraction of the plasma RNA, knowing that the blood RNA degrades rapidly after sampling. The addition of a solution such as Trizol or immediate plasma freezing at low temperature (minimum of −80 °C) allows better conservation of the integrity of the plasma RNA. In practice, this is not always possible to do in clinical routine. The second hurdle is the low detection limit of the *EML4-ALK* rearrangement by RT-PCR (around 20–25%), which also limits its use in daily practice [43].

## 5. Analysis of the Status of *ALK* in Platelets

Several studies have shown that platelets can sequester tumor RNA by taking up circulating micro-vesicles [44,45]. These tumor-educated platelets can be isolated and constitute an enriched source of tumor RNA for detection of *EML4-ALK* rearrangements. A recent study showed that this approach, while being very specific, is more sensitive than those used for plasma RNA [43]. It is noteworthy that the persistence of *EML4-ALK* rearrangements in platelets of patients treated with crizotinib is associated with tumor progression and poor prognosis [43]. Moreover, the reappearance of this fusion transcript in platelets is a sign of resistance to crizotinib and it occurs several months before progression is detected on radiological examination.

## 6. What Can Approaches such as NGS Bring to Detection of *ALK* Rearrangements in Liquid Biopsies?

In addition to the *ALK* rearrangements, other fusion transcripts of the genes for ROS proto-oncogene 1 (*ROS1*), ret-proto-oncogene (*RET*) or *NTRK* genes have been identified in 1–2% of patients with advanced stage lung cancer. These genomic alterations are liable to be associated with targeted therapies. In this context, NGS approaches were developed to identify these genomic alterations in purified free nucleic acids in blood samples [24,46,47,48]. Based on the hybrid-capture approach, these techniques not look only for rearrangements but also point mutations, insertions, deletions and variations in the number of copies. At present, the NGS techniques are being developed for use with LB and to thereby allow detection of several targeted therapeutics of interest with a single approach. However, these approaches probably still lack sufficient sensitivity to date and different ring studies will be probably useful before using them in clinical routine practice. 

## 7. Detection of ALK Mutations in Liquid Biopsies

Despite a frequent effective therapeutic response in the first months of administration of targeted therapeutics the patients with an *ALK* rearrangement relapse or progress, essentially due to the emergence of mutations in *ALK* [15]. Many different *ALK* mutations occur and can be detected by NGS analysis with free circulating tumor DNA. The detection of these mutations leads to changes in the targeted therapeutics. It is important to note that depending on the treatment used to target an *ALK* rearrangement, the resistance mutations that emerge on the *ALK* gene can be different [15]. For example, the L1196M mutation often occurs after treatment with crizotinib, G1202R after ceritinib or alectinib, F1174C after ceretinib and I1171T/N/S after alectinib treatment [15].

## 8. Advantages and Limits of the Liquid Biopsies for the Assessment of *ALK* Status

As for the detection of *EGFR* activating and resistance mutations, LB can hold specific indications and advantages compared to a tissue biopsy (Table 1). Thus, this approach is non-invasive, can be repeated easily and can be an alternative to tissue analysis for an *ALK* rearrangement. In fact, an analysis using blood can be first performed when a tissue biopsy cannot be performed (in the case of the presence of a non-biopsiable tumor site and if the patient is fragile), when the percentage of tumor cells in a tissue biopsy is too small and/or when the amount/quality of biopsy tissue is insufficient (inadequate fixation used, hypo- or hyper-fixation, cell flattening or presence of an extensive zone of necrosis). Within the context of follow-up of patients receiving targeted therapy, LB holds several advantages compared to tissue biopsy. During tumor progression of fragile patients, this non-invasive approach can investigate persistent rearrangement or detect resistance mutation onset in the *ALK* gene and thereby treatment can be adapted with respect to the observed mutation. After a change in the treatment, renewed LB can be again performed at different times to look for the appearance of new mutations of the *ALK* gene. Monitoring of patients treated with crizotinib or alectinib can also be done by liquid biopsy. The persistence or reemergence of an *ALK* rearrangement can indicate the absence of response to treatment or can predict radiological progression before the appearance of clinical symptoms. 

Despite this, the detection in blood of the *ALK* status and *ALK* mutations holds certain limits (Table 1). The detection of CTCs is not a standardized approach of daily practice and reproducibility requires good technical skills. Moreover, CTC detection techniques are not available in all institutions and thus not easily accessible to all patients. The detection of an *ALK* rearrangement in plasma or in platelets can only be performed if the pre-analytical phases are perfectly controlled (appropriate tubes to collect and package the blood, short delay for transfer to the laboratory, well established centrifugation protocols, storage of plasma or platelets at low temperature). Beyond mastering the techniques, it should be noted that the amount of CTCs or plasma RNA or RNA associated with platelets can be very variable depending on the patient and the tumor biology and the tumor mass. Thus, certain patients, even during the metastatic phase, can have a very low number of CTCs and/or a low amount of plasma RNA. In addition, it is possible that 10 mL of blood is not sufficient to isolate enough quantity of tumor RNA, particularly for NGS methods.

## 9. Conclusions

*ALK* rearrangements and *ALK* mutations can be detected with a LB. The specificity of detection is very good with CTCs, plasma and platelets. As described above, different approaches exist and the combination of these approaches might increase the chance to detect *ALK* rearrangements and *ALK* mutations. However, future efforts should be done to compare these methods in the same cohorts of patients to see the potentiality to use them simultaneously to increase the number of *ALK* positive blood samples. In the event of a positive result in blood samples, correlation with the results obtained with tissues is excellent. In contrast, the sensitivity is variable depending on the approach used, notably for techniques developed with plasma free RNA. The benefit of LB is the same as for the detection of *EGFR* mutations in blood: (i) for detection of an *ALK* rearrangement when it is impossible to perform a tissue biopsy or if the tissue sample is degraded or not exploitable; (ii) for early detection of *ALK* mutations associated with resistance to treatment leading to a rapid change in the therapeutic strategy; and (iii) for monitoring of the efficacy of treatments targeting an *ALK* rearrangement when this genomic alteration is lost or inversely reappears in blood.

The challenge now is certainly to increase the sensitivity of the blood tests for this detection and to optimize the NGS approach, combining the search for *EGFR*; rearrangements in *ALK*, *ROS1*, *RET*, and *NTKR*, but also other genomic alterations such as *BRAF*, *HER2*, and *MET*; and investigations for MSI-H in LB. This requires mastering the pre-analytical phase as soon as the blood is sampled and the development of more sensitive technologies with a minimal amount of circulating DNA/RNA. Currently, other approaches are being studied for determination of the *ALK* status in urine or with plasma microRNAs [49,50]. Finally, one of the major issues will be to propose robust and reproducible analytical tests to define the *ALK* status in blood, under the guise of quality control with accredited tests approved according to international norms [51,52].

## Figures and Tables

**Table 1:** The pros and the cons of anaplastic lymphoma kinase (*ALK*) gene analysis with a liquid biopsy in non small cell lung cancer patients.

**Pros**	
	Non-invasive method
	Easily repeatable
	Monitoring for early detection of mutation onset in the *ALK* gene
	Alternative approach to a tissue biopsy when: Presence of a non-biopsiable tumor site Patient is fragile Presence of a weak percentage of tumor cells Poor quality of extracted RNA
**Cons**	
	*ALK* status assessment in circulating tumor cells is not available routinely
	The quality of the results is strongly related to some pre analytical parameters: Volume of blood sample Tubes used for blood collection Delay for transfer to the laboratory Centrifugation procedure Temperature of plasma/platelets storage
	Large variability of amount of CTCs and free tumor RNA and platelets associated tumor RNA according to the patient
